# 

**Published:** 1859-01

**Authors:** 


### AMERICAN JOURNAL

OF

### DENTAL SCIENCE:

EDITED BY

CHAPIN A. HARRIS, M. D., D. D. S.

AND

A SNOWDEN PIGGOT, M. D.

VOL. 9—NEW SERIES.

AND VOL. 19 INCLUDING FIRST SERIES.

Surgeon Genl's Office
LIBRARY
21694
Washington, D.C.

PHILADELPHIA:
LINDSAY & BLAKISTON.
LONDON:
TRUBNER & CO., 12 PATERNOSTER ROW.
1859.

ANNEX

W
1
AM 45 M

J. W. WOODS, PRINTER,

BALTIMORE.

### TO READERS AND CORRESPONDENTS.

Communications intended for publication and exchange journals and papers, should be addressed to the EDITORS OF THE JOURNAL, Baltimore, Md. All letters relating to the BUSINESS of the Journal, or containing CASH REMITTANCES to be directed to Messrs. LINDSAY & BLAKISTON, Publishers, Philadelphia, Penn. Articles for publication should be received by the editors at least six weeks previous to the first day of the month on which the number in which it is designed for them to appear, is issued.

### *Medical and Dental Journals Received.*

1. *American Journal of Science and Arts.* Edited by PROF. B. SILLIMAN, B SILLIMAN, JR., JAMES D. DANA, ASA GRAY, and L. AGASSIZ. November. *In Exchange.*

2. *The Dental Register of the West.* Edited by J. TAFT and GEO. WATT. S. Published by order of the Mississippi Valley Association of Dental Surgeons. December. *In Exchange.*

3. *The Dental News Letter.* Edited by J. D. WHITE and J. R. MCCURDY, D. D. S. October. *In Exchange.*

4. *Boston Medical and Surgical' Journal.* Edited by W. W. MORLAND and FRANCIS MINOT, M. D. Received regularly. *In Exchange.*

5. *The British and Foreign Medico-Chirurgical Review.* Received regularly. October. *In Exchange.*

6. *The American Journal of the Medical Sciences.* Edited by ISAAC HAYS, M. D., Surgeon to Wills Hospital, &c. October. *In Exchange.*

7. *The Ohio Medical and Surgical Journal.* Edited by RICHARD L. HOWARD, M. D. Not received. *In Exchange.*

8. *The Southern Medical and Surgical Journal.* Edited by DRS. HENRY F. and ROBERT CAMPBELL. October, November and December. *In Exchange.*

9. *The New York Medical Gazette, and Journal of Health.* Edited by D. M. REESE, M. D., LLD. October, November and December. *In Exchange.*

10. *The New York Journal of Medicine and the Collateral Sciences.* Edited by STEPHEN SMITH, M. D. November. *In Exchange.*

11. *The North American Medico-Chirurgical Review.* Edited by Drs. GROSS and RICHARDSON. November. *In Exchange.*

12. *The Nashville Journal of Medicine and Surgery.* Edited by W. R. BOWLING, M. D., assisted by R. C. FOSTER and GEORGE S. BLACKIE, M. D. October, November and December. *In Exchange.*

13. *The New Jersey Medical Reporter, and Transactions of the New Jersey Medical Society.* Edited by JOSEPH PARRISH, M. D. Received regularly every week. *In Exchange.*

14. *The Edinburgh Medical and Surgical Journal.* Not received. *In Exchange.*

15. *The Cincinnati Lancet and Observer.* Edited by Drs. G. Mendenhall, John A. Murphy and Ed. B. Stevens. *In Exchange.*

16. *The Chicago Medical Journal.* Edited by Drs. N. S. Davis and W. H. Byford. *In Exchange.*

17. *The British Journal of Dental Science. In Exchange.*

18. *The New Hampshire Journal of Medicine. In Exchange.*

19. *The Charleston Medical Journal aud Review.* Edited by D. J. Cain, M. D., and F. Peyre Porcher, M. D. *In Exchange.*

20. *The Buffalo Medical Journal.* Edited by Austin Flint, M. D. *In Exchange.*

21. *Dublin Quarterly Journal.* November. *In Exchange.*

22. *The Dublin Medical Press.* Received very irregularly. *In Exchange.*

23 *The Peninsular Journal of Medicine and the Collateral Sciences.* Edited by E. Andrews, A. M., M. D. etc. *In Exchange.*

24. *Virginia Medical and Surgical Journal.* Edited by Drs. George A. Otis and H. L. Thomas. Not received. *In Exchange.*

25. *The St. Louis Medical and Surgical Journal.* Edited by L. Linton, M. D., and Wm. M. McPheeters, M. D. *In Exchange.*

26. Der Zahnarzt. *Das Neueste und Wissenswürdigste des In—und Auslandes über* Zahnheilkunde. *Redakteur ;* C. Schmedicke, *prakt. Zahnarzt zu* Berlin. *In Exchange.*

27. *Medical Times and Gazette.* Received regularly. *In Exchange.*

28. *Annales de la Sociedad de Medicina Montevideana.* In Exchange.

29. *L'Art Dentaire.* Edited by MM. Fowler and Preterre. *In Exchange.*

30. *Quarterly Journal of Dental Science.* London. *In Exchange.*

31. *The New Orleans Medical News and Hospital Gazette.* Edited by D. Warren Brickell, M. D , and E. D. Fenner, M. D. Received regularly. *In Exchange.*

32. *American Dental Review.* Edited by Dr. A. M. Leslie. *In Exchange.*

*American Dental Convention.*—The following report furnished us by Dr. Allport, Chairman of the Executive Committee, shows the order of the business for the next Convention. We trust it will be well attended, and that the subjects enumerated in the report will receive the attention their importance merits. Work enough is here laid out to occupy it fully one week. EDS.

*Report of the Executive Committee.*—The Executive Committee appointed at the last "American Dental Convention," would respectfully report the following as the order of business for the next Convention, which is to meet at "Niagara Falls" on the first Tuesday in August, 1859, at 10 o'clock, A. M.

1st. Reading and correcting report of the last meeting.
2d. Treasurer's report.
3d. Report of Committees.
4th. Election of Officers.
5th. The retiring President's Address.
6th Installation of Officers.
7th. Appointing Committees.

*Discussions.*

1st. When, and under what circumstances should teeth be extracted, to secure a correct development of the permanent teeth?

2d. Mechanical appliances and forceps, to correct irregularities of the teeth.

3d. Under what circumstances, and in what manner should teeth be filed, to remove superficial decay, or preparatory to filling, to leave them the most secure from future decay?

4th. Filling Teeth.

5th. Mechanical Dentistry.

6th. Treatment of Diseases of the Antrum.

7th. Professional Intercourse.

8th. Miscellaneous.

The reading of original Essays on the various topics under discussion will be first in order under their appropriate heads.

The Committee would respectfully suggest to all who expect to be present, the benefit as well as the *propriety* of preparing notes, (to be read before the Convention) of their experience and observation on the various subjects named for discussion. Let these notes be carefully prepared, *short and to the point*. In this way a large amount of valuable information may be collected for publication, and thus be put on record for the benefit of the profession at large.

W. W. Allport, Frank Fuller,
W. M. Wright, A. M. Leslie.
W. D. Stone,

---

*To Correspondents.*—We are always happy to answer inquiries, to the best of our ability, made by members of the dental profession, but we have received so many letters within the last few months, asking our opinion with regard to the value of the Cheoplastic Process of mounting teeth, as compared with the other methods now in use, that we can scarcely find time to reply to each one separately. To the numerous inquiries, therefore, upon this subject, we beg leave to state that the following extract taken from the last edition of the senior editor's Principles and Practice of Dental Surgery, embodies in few words, the information which he possesses in relation to the matter.

"Among the peculiar advantages claimed for the Cheoplastic over the other methods now practiced, of mounting artificial teeth, are perfect accuracy of adaptation of the base to the plaster model—metallic castings not being used in this process—and greater practical usefulness and durability. It can also be done in less time, and the material used for the base in this process is less expensive—it being an alloy, the precise composition of which we have never taken pains to ascertain, as it can be obtained from the manufacturer, and at most of the dental depots, of a better quality, we presume, and at a lower price than it can be made for in small quantities. It is, however, composed principally of tin, silver and bismuth, with a small trace of antimony.* The alloy imparts no taste whatever to the mouth, and its purity, so far as its capability of resisting the action of the secretions of the buccal cavity is concerned, is said to be fully equal to that of eighteen carat gold. The lustre of it, after being worn some weeks, becomes slightly dimmed, but is immediately restored by placing it in a strong solution of caustic potash. This is the only change we have ever observed, and we have seen it after having been worn in the mouth nearly two years.

"This method of mounting teeth has only been practiced since the fall of 1855, that is, with the above mentioned alloy, and it was not made known to the profession generally until February, 1857. Since this time it has been adopted and practiced by nearly three hundred, and among the number are many of the most skillful and respectable practitioners of the United States. Thus far, we believe it has realized the expectations of its most zealous advocates, and judging from the testimony of others, as well as from results which have come under our own observation, the use of it seems likely, in a very short time, to become general."

*As the right to manufacture the alloy, as well as the entire process, has been secured to the inventor, Dr. A. A. Blandy, by letters patent, the exact composition may be seen in the specification which accompanied his application for the patent.

